# Can Leaders Prevent Technology From Backfiring? Empowering Leadership as a Double-Edged Sword for Technostress in Care

**DOI:** 10.3389/fpsyg.2021.702648

**Published:** 2021-06-23

**Authors:** Robin Bauwens, Marith Denissen, Jeske Van Beurden, Martine Coun

**Affiliations:** ^1^Department of Human Resource Studies, Tilburg University, Tilburg, Netherlands; ^2^Faculty of Management Sciences, Open University, Heerlen, Netherlands

**Keywords:** technostress, emotional exhaustion, quality of care delivered, conservation of resources theory, empowering leadership, techno-overload, techno-complexity, techno-invasion

## Abstract

**Purpose:** Recent studies have called for more contextual studies of technostress and the role leaders can have in this experience. While technostress is an increasingly prevalent and severe phenomenon in care professions, limited studies have addressed its potential negative consequences for employee well-being and quality of care delivered in this sector or, more importantly, examined how the adverse consequences of technostress could be mitigated. Therefore, the present study addresses this gap by investigating how technostress in childcare affects quality of care delivered via emotional exhaustion and what influence empowering leadership plays in this relationship.

**Design/methodology approach:** Incorporating the views of 339 Dutch childcare workers, this study tests a model in which technostress influences quality of care delivered, mediated by emotional exhaustion and moderated by empowering leadership.

**Findings:** Results confirm that techno-invasion and techno-overload predict higher emotional exhaustion and lower quality of care delivered among childcare workers. Empowering leadership reduced the influence of techno-invasion on emotional exhaustion but strengthened the influence of techno-overload.

**Originality/value:** Our results provide childcare organizations with relevant information on the increasing use of ICT that influences both childcare workers' well-being and quality of care they deliver. Important implications are suggested for leadership geared at stimulating employees' responsibility and accountability for different dimensions of technostress.

## Introduction

In recent years, childcare organizations have made significant investments in information and communication technology (ICT) to improve their professional services (Denissen, 2020). A survey from 2018 demonstrates that the majority of Dutch childcare organizations engaged in some kind of ICT innovation in the last 12 months. Most popular were the digitalization of internal work processes (52%), like personnel planning, invoicing and logistics services, followed by contact with clients (45%), for example through social media or “digital parent environments” and the use of e-learning for staff (33%) (FCB, 2018). On the one hand, ICT provides substantial benefits to childcare organizations, such as quality monitoring and enhanced service to clients (Yost and Fan, 2014). On the other hand, ICT can also make work more complex, intensive and could even induce technostress, which could prevent ICT's benefits from materializing (Bauwens et al., 2020; Molino et al., 2020). Technostress refers to stress resulting from individuals' inability to cope with technology, like ICT, and its associated changes (Brod, 1982; Ragu-Nathan et al., 2008). This is problematic, as it yields a variety of negative consequences for individual job outcomes (Tarafdar et al., 2015; La Torre et al., 2019).

To date, technostress research has been mostly restricted to contexts like government, industry (Ayyagari et al., 2011) and education (Wang W. et al., 2020; Penado Abilleira et al., 2021). However, technostress is an increasingly prevalent and severe phenomenon in care professions (Califf et al., 2015; Fagerström et al., 2017). Childcare workers in particular constitute a group whose work-related well-being is continuously challenged, since they are regularly confronted with physical complaints (e.g., noise, musculoskeletal complaints), emotional demands (e.g., emotional responses from children and parents), and operate in a highly regulated environment (Decker et al., 2002; Løvgren, 2016; Koch et al., 2017). This situation has potentially been exacerbated by recent technological developments (Denissen, 2020), but also by the current COVID-19 pandemic, as childcare workers found themselves at the frontline (Bradley and Chahar, 2020). To resolve the challenges of technostress for employees, scholars inspired by e-leadership literature point to the role of leaders (Cortellazzo et al., 2019; Bartsch et al., 2020; Iannotta et al., 2020). Leaders play an important part in regulating how employees' stress experiences affect individual job outcomes (Zhang et al., 2014; Wang X. et al., 2020). However, with a few notable exceptions, technostress research has seldomly addressed leaders' influence in mitigating the adverse consequences of technology (Salanova et al., 2013; Turel and Gaudioso, 2018; Spagnoli et al., 2020). In addition, limited studies have examined the potential negative consequences of technostress in childcare. Let alone, how such potential adverse consequences could be mitigated by leaders.

The present study addresses this hiatus examining how technostress, more specifically stressors like techno-overload, techno-invasion and techno-complexity, affect the quality of care delivered among childcare workers, by focusing on the mediating role of emotional exhaustion. In addition, we investigate how these relations are affected by leadership. Emotional exhaustion is a key predictor of burnout and describes employees' feeling of being emotionally drained by work (Maslach et al., 1986; Wright and Cropanzano, 1998). Furthermore, quality of care delivered, in absence of a generally accepted definition, refers to the extent that effective care is provided to those who require it (Humphries et al., 2014). Not only is quality of care the main “output” of childcare organization, its importance is also underscored by several laws and regulations, like the Innovation and Quality Act in the Netherlands^1^ (i.e., *Wet Innovatie en Kwaliteit Kinderopvang*). In linking technostress to quality of care delivered via emotional exhaustion, the present study taps into Conservation of Resources theory (COR; Hobfoll, 2001). COR theory advances that employees have limited resources (e.g., social support, time, technological literacy). Resources can help employees in coping with stressful experiences but are typically also depleted by confrontations with (techno)stress. Conversely, facilitating factors like leadership can also help to replenish such resources and let employees overcome stressful experiences (Turel and Gaudioso, 2018; Lutz et al., 2020).

A particular leadership approach that is concerned with employees' resources and aligns well with the logic of COR-theory is empowering leadership (Kim and Beehr, 2021). Empowering leadership encompasses leader behaviors that encourage and support employees' autonomy, participative decision-making, and meaning of work (Arnold et al., 2000; Ahearne et al., 2005; Audenaert and Decramer, 2016). As technologies like ICT limit leaders' direct opportunities for control and make work more complex and unpredictable, employees are increasingly required to demonstrate more responsibility, self-leadership, and own solutions for work-related problems. Therefore, empowering leadership, which reinforces such proactive employee behaviors, presents itself as a promising leadership style to help employees overcome challenges in ICT contexts (Hill and Bartol, 2016; Coun et al., 2021). Furthermore, past research underscores the merits of empowering leadership in care settings, where employees typically have to balance multiple job demands and resource constraints (Audenaert et al., 2020; Bauwens et al., 2021). Based on this reasoning, we expect empowering leadership to moderate the relationships between technostress, emotional exhaustion and quality of care delivered.

In testing these relationships, we make a two-fold contribution. Our first contribution is to technostress literature. Like general stress, technostress is context specific La Torre et al. (2019). By expanding the knowledge on technostress in care professions (Califf et al., 2015; Fagerström et al., 2017), this study furthers the work of Tarafdar et al. (2015) on the contextualization of technostress, demonstrating that this phenomenon is not limited to high-tech or business environments (Wang W. et al., 2020), but also affect employees in sectors that are currently making significant ICT investments to improve their professional services (Yost and Fan, 2014) and have traditionally received less attention in this regard. Second, by accounting for empowering leadership we contribute to the growing literature on this leadership approach (Dinh et al., 2014; Cheong et al., 2019) and extend the emergent body of research on the importance of leadership in the contemporary ICT-infused workplace (Cortellazzo et al., 2019; Gardner et al., 2020; Iannotta et al., 2020). By not only focusing on the adverse consequences of technostress, but also on how these can be mitigated by leadership, this study contributes to calls for more “proactive” studies on technostress, which could inspire more effective interventions for reducing technostress within organizations (Parker and Grote, 2020).

### Technostress

Technostress was first coined by Brod (1982), defining it as an adaptation problem “caused by an inability to cope with the new computer technologies in a healthy manner” (p. 16). While many authors have sought to broaden or adjust this definition (e.g., Weil and Rosen, 1997; Ragu-Nathan et al., 2008; Salanova et al., 2013), there seems to be an agreement that technostress is essentially a negative, technology-induced psychological state that has an adverse impact on people's attitudes and behaviors (cf. La Torre et al., 2019). Technostress seems increasingly prevalent and severe among care professionals. While several sectors have witnessed increases in the adoption and intensity of ICT in their work over the past few years, such transformations have been relatively abrupt in professional care, compared to government and business environments, where such transformations have occurred more gradually (Califf et al., 2015; Fagerström et al., 2017). In particular, many childcare organizations in recent years saw the introduction of digital parent environments (i.e., web-based page or app to share information about the child with their parents), the emergence of social media pages, and the digitization of administrative rules and procedures among other technological developments (Yost and Fan, 2014; Bauwens and Meyfroodt, 2021). Consequentially, childcare workers are exposed to ICT-induced stressors, i.e., techno-stressors. Examples of such techno-stressors include stress due to information overload (techno-overload), the invasion of technology to the private sphere (techno-invasion), the sheer complexity of technology (techno-complexity), constant changes in hardware and software (techno-uncertainty) and/or concerns over future employment (techno-insecurity; Tarafdar et al., 2010). While some have sought to broaden (Fischer et al., 2021) or challenge (Hu et al., 2021) this conceptualization of techno-stressors, scholars like Molino et al. (2020) and Spagnoli et al. (2020) recently made a convincing case for a more parsimonious approach, which focuses on techno-overload, techno-invasion and techno-complexity as three predominant stressors in contemporary jobs. For example, childcare workers might struggle with the techno-complexity and techno-overload of information (cf. Shu et al., 2011; Harris et al., 2015) that administrative systems, parent environments or even simple WhatsApp groups generate. Alternatively, work-related ICT use might find its way into the private sphere (cf. Schlachter et al., 2018; Bauwens et al., 2020) in the form of texts and notifications from colleagues, parents or bosses and thereby prevent mental recovery from work.

### The Mediating Role of Emotional Exhaustion

Childcare workers are an employee group whose health-related well-being is continuously at risk, a situation that is potentially exacerbated by recent technological developments. Being subjected to high physical demands, rule demands and emotional labor, childcare workers are susceptible to episodes of emotional exhaustion (Decker et al., 2002; Løvgren, 2016; Koch et al., 2017). Emotional exhaustion is a key facet of burnout and refers to a chronic state of emotional and physical fatigue (Maslach et al., 2001). Past research has linked emotional exhaustion to stress at work by adopting the theoretical lens of COR theory. This theory advances that a lack of resources will lead to defensive attempts to preserve the remaining resources (Hobfoll, 2001). According to Hobfoll (2001), resources are characteristics, conditions or objects that are valued by employees and help them to achieve or protect other valued resources. COR theory states that when valued resources are threatened or are not adequately replenished, this incites negative job outcomes, like emotional exhaustion (Srivastava et al., 2015; Kilroy et al., 2020). Applied to the present context, childcare workers consume valuable resources when dealing with techno-stressors. Therefore, childcare workers that are confronted with such stressful situations, see their emotional and physical resources depleted. This leaves them with insufficient resources to deal with their other job demands (Ghislieri et al., 2017) and ultimately renders them more vulnerable to develop burnout-related symptoms, like emotional exhaustion (Wright and Cropanzano, 1998; Hobfoll and Freedy, 2017). Empirical studies on technostress and emotional exhaustion seem to confirm this line of reasoning (Brown et al., 2014; Srivastava et al., 2015; Gaudioso et al., 2017; Califf and Brooks, 2020). We therefore predict that:

H1: Technostress, in terms of techno-overload, -invasion, and -complexity, is positively related to emotional exhaustion.

COR theory asserts that employees' initial loss of resources will lead to future losses, resulting in so-called “downwards loss spirals” (Hobfoll, 2001). Accordingly, experiencing technostress might deplete employees' resources, culminating in emotional exhaustion and further eroding employees' resources in a vicious cycle. COR theory predicts that employees who experience such continued resource losses will prioritize how they use their remaining resources. Confronted with technostress, employees might experience emotional exhaustion due to depleted resources. Consequentially, those employees might shift their priories to coping with this exhaustion, rather than spending such resources to their job performance (Hobfoll, 2001; Hobfoll and Freedy, 2017).

In childcare organizations, one of the main aspects of performance is quality of care delivered, or the extent to which effective care is provided to those who require it (Humphries et al., 2014). In person care environments, there are typically limits to the extent to which ICT tasks and care duties are compatible with one another (Califf et al., 2015). For example, changing diapers or feeding and playing with children is often difficult to combine with ICT tasks like handling administration and communicating with parent through social media. In other words, COR theory and its “loss spirals” lead to suggests that emotional exhaustion could act as a mechanism through which technostress affects quality of care delivered. This in line with the work of Karatepe and Uludag (2008), who previously demonstrated a mediation of emotional exhaustion between general stress and job performance. In addition, there is empirical support for linking technostress to both emotional exhaustion (Brown et al., 2014; Srivastava et al., 2015; Gaudioso et al., 2017) and job performance (Tarafdar et al., 2010, 2015; Brooks and Califf, 2017; Wang X. et al., 2020). The latter also show close relations in prior studies (Wright and Cropanzano, 1998; Humphries et al., 2014; Alves and Guirardello, 2016). Therefore, the following hypotheses are proposed:

H2a: Emotional exhaustion is negatively related to quality of care delivered.
H2b: The negative relationship between technostress and quality of care delivered is mediated by emotional exhaustion.

### The Moderating Role of Empowering Leadership

COR theory is not only concerned with how employees' resources are depleted, but also with how such resources can be replenished by certain facilitating factors (Hobfoll, 2001). Leadership constitutes an important facilitating factor for replenishing employees' resources. While traditionally regarded as a resource in itself, contemporary scholars progressively draw attention to leaders' influence on the allocation and impact of resources among employees (Schaufeli, 2015). Therefore, the influence of leadership on employees' experiences at work—and its integration in COR theory—is increasingly regarded as important in its own right (Hobfoll et al., 2018). A particular leadership approach that is concerned with strengthening employees' resources is empowering leadership. Empowering leaders display non-directive leader behaviors that foster employees' autonomy, participative decision making and problem-solving behavior (Arnold et al., 2000; Ahearne et al., 2005; Audenaert and Decramer, 2016). Consistent with the logic of resource replenishment in COR theory, empowering leaders strengthen employees' resource base through coaching, promoting their self-development, expressing confidence in their abilities, and stimulating them to broaden their scope of potential solutions for given problems by exploring opportunities and alternatives (Windeler et al., 2017; Kim and Beehr, 2021). Therefore, empowering leadership presents a suitable leadership style for challenging work environments, like person care (Audenaert et al., 2020; Liu et al., 2021) and those characterized by ICT (Hill and Bartol, 2016; Windeler et al., 2017; Coun et al., 2021). When empowering leadership is relatively high, childcare workers might be stimulated to look for potential solutions that limit the impact of techno-stressors on emotional exhaustion and preserve the quality of care delivered (cf. Turel and Gaudioso, 2018). For example, empowering leaders can prompt childcare workers to set more strict work-home boundaries for themselves and/or to make shared decisions on whether and to what extent ICT is used in the childcare facility (e.g., limiting answering mails or updating parent environments to certain slots of the day or designating such responsibilities to certain colleagues), thereby reducing the influence of techno-invasion. Alternatively, empowering leaders might encourage employees to proactively help colleagues who struggle with ICT usage or with managing the ongoing information overload (e.g., teaching peer-to-peer, coming up with a role division and clear workflow), thereby reducing techno-complexity and techno-overload. However, when empowering leadership is relatively low, employees might lack the incentives to seek solutions and engage in shared decision making to address ICT issues, rendering the impact of technostress on their well-being and performance more severe. This logic is supported by studies suggesting that empowering leadership reduces the influence of specific (techno)stressors on employee outcomes. For example, Windeler et al. (2017) reported that empowering leaders reduce the influence of techno-complexity, while Kim and Beehr (2020) observed such leaders to mitigate negative technological spillovers from work to home. Additional empirical support underpins that leaders that build employees' resource base (Harris et al., 2015) and stimulate their participative decision making (Turel and Gaudioso, 2018) significantly reduce the influence of technostress(ors) on employee outcomes, like emotional exhaustion and job performance. Therefore, we hypothesize:

H3: The indirect negative effect of technostress on quality of care delivered via emotional exhaustion is weaker for employees with an empowering leader.

## Materials and Methods

### Participants and Procedure

Data were collected in September and October 2020 through an online self-reported questionnaire (Qualtrics). The questionnaire was sent to the directors of the 9,056 childcare facilities registered in the National Childcare Registry^2^ (i.e., Landelijk Register Kinderopvang), which jointly employ about 95,000 childcare workers. While the COVID-19 pandemic was ongoing at the time, childcare workers in the Netherlands continued to work onsite during the period of the data collection.^3^ The nature of their job also did not allow for telework. Since data collection through survey is susceptible to common method bias (CMB), we followed earlier recommendations to mitigate such bias. For example, separating variables in the questionnaire to create a psychological lag time and stressing voluntary and anonymous participation (George and Pandey, 2017). Accompanying the survey was a mail and cover page that gave more information about the study, stressing anonymity and that the data would only be used for study purposes. Informed consent was obtained from the respondents through a digital form in the questionnaire, in which they acknowledged their voluntary participation in the study, their right to redraw and agreed with the digital processing and storage of their answers for scientific purposes. The Ethics Review Board of the first author's institution gave permission for this study and confirmed that the rights and privacy of study participants were sufficiently accounted for (nr. EC-2019.76). In total, 339 childcare workers completed the questionnaire. In terms of age and gender, the characteristics of childcare workers in our sample resembled the characteristics of the Dutch childcare workforce (CBS, 2020). Respondents' age ranged from 19 to 64 (on average 40.66 years). Most of the respondents were female (96.7%) and worked part-time (68.4%) on a fixed contract (85.8%). Concerning the professional use of ICT, most respondents indicated its use for contact with parents (91.2%), followed by administration (82.0%) and monitoring childcare capacity and personnel planning (80.5%). To a lesser extent, childcare workers also reported the professional use of ICT to communicate with their supervisor (71.7%) or colleagues (80.5%). In addition, some respondents indicated to use ICT for other purposes, for example to access child-monitoring systems, set-up learning activities for children or engage with official authorities (e.g., GGD or Municipal Health Services).

### Measures

All measures were derived from prior-validated scales and administered in Dutch after a forth-back translation procedure. Answers were scored on a 7-point scale (1 = not at all; 7 = to a very large extent).

*Technostress* was measured using the eleven-item scale by Molino et al. (2020). This scale presents a validated, parsimonious alternative to original scale by Ragu-Nathan et al. (2008) and focusses on the three main techno stressors in contemporary jobs: techno-overload (four items including “I am forced by technology to work much faster”), techno-invasion (three items including “I feel my personal life is invaded by this technology”) and techno-complexity (four items including “I do not find enough time to study and upgrade my technology skills”). Cronbach's alpha was 0.882 for the overall scale and 0.919, 0.813 and 0.866 for the respective subdimensions.

*Empowering Leadership* was measured using the six-item scale by Pearce and Sims (2002). A sample item is “My supervisor encourages me to seek solutions without his/her direct input.” Cronbach's alpha was 0.858. *Emotional exhaustion* was measured using the five-item scale by Schaufeli et al. (1996). An example item is “I feel tired when I get up in the morning and have another working day ahead of me.” Cronbach's alpha was 0.908.

*Quality of care delivered* was assessed by the three-scale from (Aiken et al., 2002). Respondents were asked to indicate the extent to which they agreed with each statement in the scale, reflecting practices that relate to their job performance within their organization. An example item is “During my last shift high quality care was provided to the children” Cronbach's alpha was 0.687.

*Control variables* were included for gender (0 = female, 1 = male), age (in years) and job status (i.e., fulltime vs. parttime employment and fixed vs. temporary employment), since past research suggests that younger workers are more susceptible to burnout, but also points to mixed effects of gender and job status for burnout, controlling for these variables is necessary (Kroon et al., 2009; Kilroy et al., 2020).

### Data Analysis

Analyses were conducted in R with the auxiliary packages Lavaan (Rosseel, 2012) and semTools (Jorgensen et al., 2018). We employed structural equation modeling (SEM) following the recommended two-step procedure (Kline, 2011). We first tested the measurement model with confirmatory factor analysis, followed by the paths between the latent variables in the structural model. Latent moderated structural equation modeling (LMS) was used to assess the interactions and conditional (indirect) effects, which better accounts for measurement errors compared to product indicators (Feng et al., 2020).

## Results

### Preliminary Analyses

CFA was performed to test the measurement model. An overview of the models and fit indices can be consulted in Table 1. We started from a four-factor model (i.e., technostress as one dimension, empowering leadership, emotional exhaustion, quality of care delivered), which we contrasted with a one-factor model to detect potential CMB, as well as a six-factor model (i.e., technostress as three dimensions, empowering leadership, emotional exhaustion, quality of care delivered). The four-factor model showed good fit with the data (χ^2^ = 549.720, df = 266, CFI = 0.936, RMSEA = 0.058, SRMR = 0.056). The one-factor model fitted the data significantly worse, suggesting CMB is no considerable concern (Δχ^2^ = 1736.082, Δdf = 6, *p* < 0.001). However, the six-factor model with three-dimensional technostress presented a significant improvement over the four-factor model and better representation of the collected data (Δχ^2^ = 665.132, Δdf = 9, *p* < 0.001). Average variance extracted (AVE) surpassed 0.50, except for quality of care delivered (AVE = 0.430), which was not deemed problematic as its composite reliability was satisfactory (CR = 0.694). Furthermore, all items loaded significantly on their hypothesized factors (range 0.493–0.920). Therefore, the six-factor model was used as a basis to test the subsequent structural models. In line with COR theory, we started from a full mediation model, which we contrasted with a partial mediation model including both direct and indirect paths from technostress dimensions to quality of care delivered, each of them moderated by empowering leadership. Note that these models have much larger degrees of freedom due to the use of LMS (Feng et al., 2020). The full mediation model demonstrated a good fit (χ^2^ = 3453.429, df = 3,363, CFI = 0.996, RMSEA = 0.009, SRMR = 0.083), while the partial mediation model fitted the data significantly worse (Δχ^2^ = 1063.885, Δdf = 964, *p* < 0.050). Therefore, the full mediation model was retained for hypothesis testing.

**Table 1:** Models and fit indices.

	**χ^2^**	**df**	**CFI**	**RMSEA**	**SRMR**
**Measurement models**					
One-factor model (common method bias)	2942.108	275	0.397	0.176	0.161
Four-factor model (hypothesized, onedimensional technostress)	1206.026	269	0.788	0.106	0.089
Six-factor model (three-dimensional technostress)	540.894	260	0.936	0.059	0.053
**Structural models**					
Full moderated mediation	3453.429	3363	0.996	0.009	0.083
Partial moderated mediation	4517.314	4327	0.993	0.012	0.089

*CFI, comparative fit index; RMSEA, root mean square error of approximation; SRMR, standardized root mean square residual*.

### Descriptive Statistics and Correlations

Table 2 presents the descriptive statistics and correlations. In line with the hypotheses, the correlations show that techno-overload, techno-invasion and techno-complexity are associated with higher emotional exhaustion (respectively *r* = 0.353, *p* ≤ 0.010; *r* = 0.361, *p* ≤ 0.010; *r* = 0.217, *p* ≤ 0.010) and lower quality of care delivered (respectively, *r* = −0.229, *p* ≤ 0.010; *r* =−0.181, *p* ≤ 0.010; *r* = −0.223, *p* ≤ 0.010). Empowering leadership was negatively related to emotional exhaustion (*r* = −0.194, *p* ≤ 0.010) and positively to quality of care delivered (*r* = 0.248, *p* ≤ 0.010), while the latter two variables were also related (*r* = −0.300, *p* ≤ 0.010). In addition, gender (female) was positively related to techno-complexity (*r* = 0.184, *p* ≤ 0.010), while age positively correlated with reports of techno-complexity (*r* = 0.397, *p* ≤ 0.010) and empowering leadership (*r* = 0.160, *p* ≤ 0.010). Furthermore, fulltime employment negatively correlated with techno-overload and techno-complexity (respectively, *r* = −0.146, *p* ≤ 0.010; *r* = −0.139, *p* ≤ 0.010) while temporary employment was negatively correlated with techno-complexity (*r* = −0.168, *p* ≤ 0.010).

**Table 2:** Descriptive statistics and correlations (*N* = 339).

		**Mean**	**SD**	**1**	**2**	**3**	**4**	**5**	**6**	**7**	**8**	**9**	**10**
1	Gender	0.967	0.179										
2	Age	40.660	11.929	−0.046									
3	Fulltime employment	0.316	0.465	−0.09	−0.198^**^								
4	Temporary employment	0.142	0.350	0.028	−0.328^**^	−0.112^*^							
5	Overload	4.854	1.700	0.095	0.016	−0.146^**^	−0.011						
6	Invasion	3.948	1.931	0.041	−0.032	−0.087	−0.025	0.608^**^					
7	Complexity	2.969	1.308	0.184^**^	0.397^**^	−0.139^*^	−0.168^**^	0.362^**^	0.285^**^				
8	Empowering leadership	5.287	0.939	0.012	0.160^**^	0.021	−0.046	−0.191^**^	−0.218^**^	−0.037			
9	Emotional exhaustion	2.402	1.274	0.033	−0.09	0.029	−0.077	0.353^**^	0.361^**^	0.217^**^	−0.194^**^		
10	Quality of Care Delivered	5.892	0.842	−0.072	−0.001	0.134^*^	−0.072	−0.229^**^	−0.181^**^	−0.223^**^	0.248^**^	−0.300^**^	

* *p < 0.05*,

** *p < 0.01*.

### Hypothesis Testing

**Figure 1:**
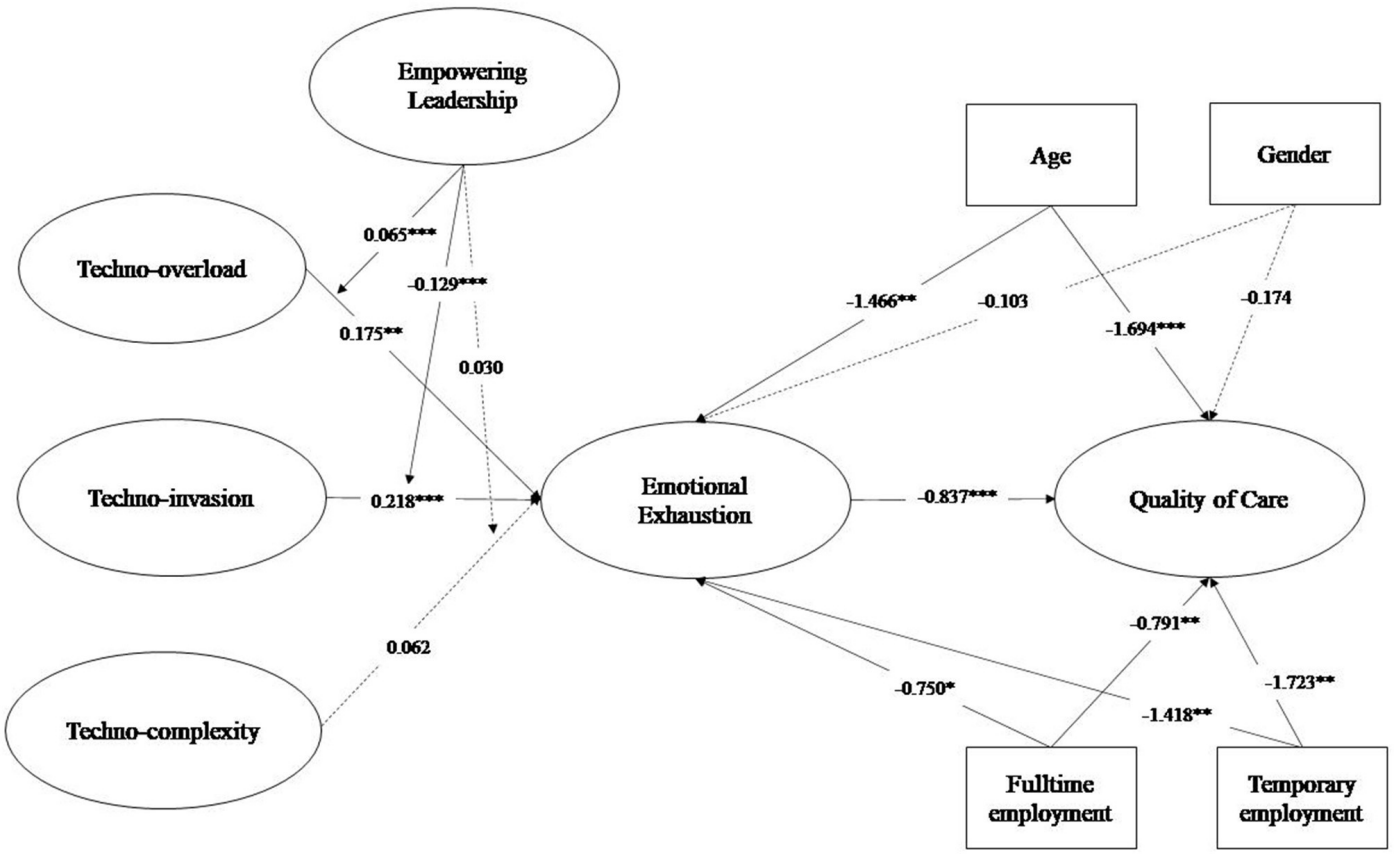
Path model.

Table 3 reports the paths from the structural model, which are also graphically depicted in Figure 1. The results show that techno-overload and techno-invasion predict emotional exhaustion (respectively *B* = 0.175, *p* ≤ 0.001; *B* = 0.218, *p* ≤ 0.001), but not techno-complexity (*B* = 0.062, *p* = 0.051). This partially supports our first hypothesis (H1). In accordance with the second set of hypotheses (H2a, H2b), emotional exhaustion also predicted quality of care delivered (*B* = −0.837, *p* ≤ 0.001). Furthermore, in line with H3a, the interaction between techno-invasion and empowering leadership was significantly related to emotional exhaustion (*B* = −0.129, *p* ≤ 0.001). This was also the case for the interaction between techno-overload and empowering leadership, but not in the hypothesized direction (*B* = 0.065, *p* ≤ 0.050).

**Table 3:** Structural paths (*N* = 301).

**Path**	**B**	**SE**	**95% CI**	***p***
**Direct effects**				
Gender → Emotional exhaustion	−0.103	1035	[−2.721; 1.337]	0.504
Age → Emotional exhaustion	−1.466	0.049	[−0.241; −0.048]	0.003^**^
Fulltime employment → Emotional exhaustion	−0.750	0.781	[−3.366; −0.304]	0.019^*^
Temporary employment → Emotional exhaustion	−1.418	2.344	[−11.177; −1.988]	0.005^**^
Techno-overload → Emotional exhaustion	0.175	0.039	[0.051; 0.202]	<0.001^***^
Techno-invasion → Emotional exhaustion	0.218	0.039	[0.064; 0.218]	<0.001^***^
Techno-complexity → Emotional exhaustion	0.062	0.035	[−0.000; 0.138]	0.051
Empowering leadership → Emotional exhaustion	−0.159	0.030	[−0.247; −0.130]	<0.001^***^
Empowering leadership × Techno-overload → Emotional exhaustion	0.065	0.022	[0.001; 0.089]	0.043^*^
Empowering leadership × Techno-invasion → Emotional exhaustion	−0.129	0.021	[−0.117; −0.034]	<0.001^***^
Empowering leadership × Techno-complexity → Emotional exhaustion	0.030	0.022	[−0.011; 0.075]	0.144
Gender → Quality of Care Delivered	−0.174	0.684	[−1.975; 0.704]	0.353
Age → Quality of Care Delivered	−1.694	0.023	[−0.137; −0.045]	<0.001^***^
Fulltime employment → Quality of Care Delivered	−0.791	0.394	[−1.827; −0.284]	0.007^**^
Temporary employment → Quality of Care Delivered	−1.723	1.157	[−6.632; −2.095]	<0.001^***^
Emotional Exhaustion → Quality of Care Delivered	−0.837	0.040	[−0.535; −0.378]	<0.001^***^
**(Conditional) indirect effects**				
Techno-overload → Emotional exhaustion → Quality of Care Delivered	−0.052	0.017	[−0.086; −0.019]	0.002^**^
Techno-invasion → Emotional exhaustion → Quality of Care Delivered	−0.075	0.022	[−0.118; −0.032]	<0.001^***^
Techno-complexity → Emotional exhaustion → Quality of Care Delivered	−0.029	0.016	[−0.061; 0.002]	0.070

* *p < 0.05*,

** *p < 0.01*,

*** *p < 0.0001*.

**Figure 2:**
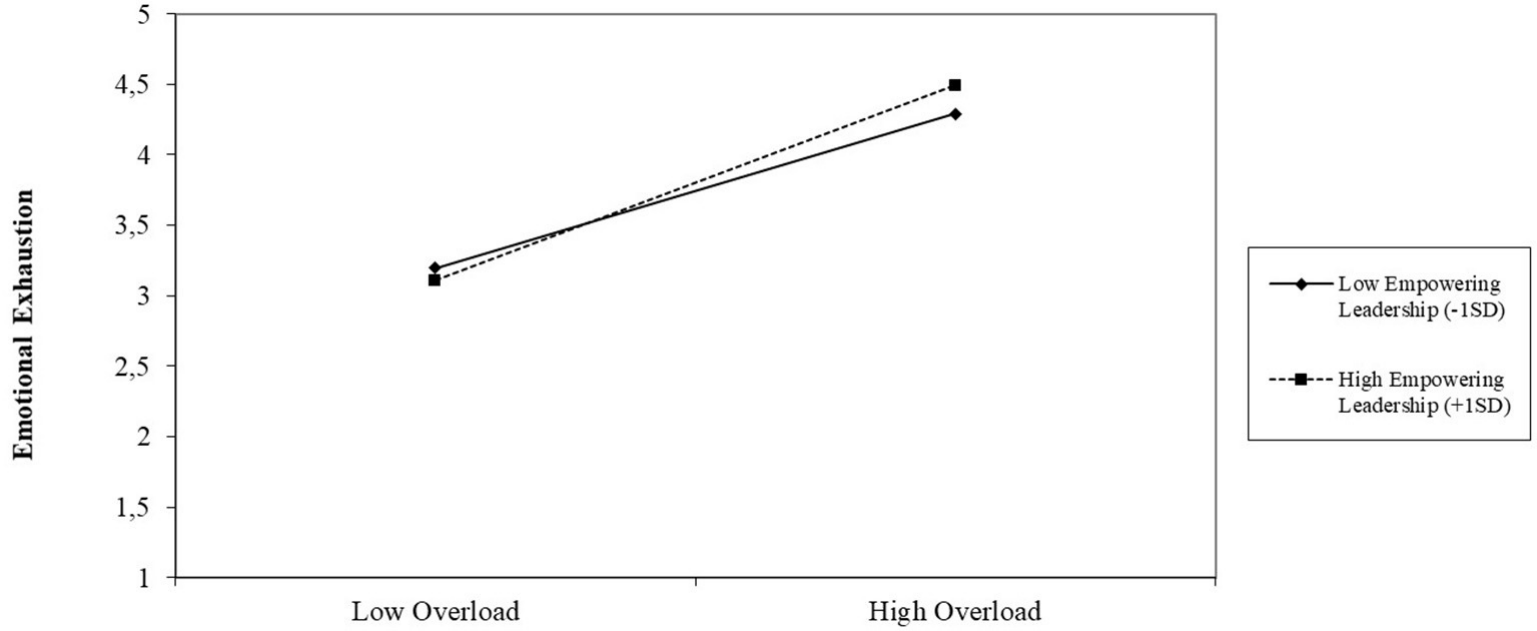
Interaction plot for the relation between techno-invasion and emotional exhaustion under high (+1SD) and low (−1SD) empowering leadership.

**Figure 3:**
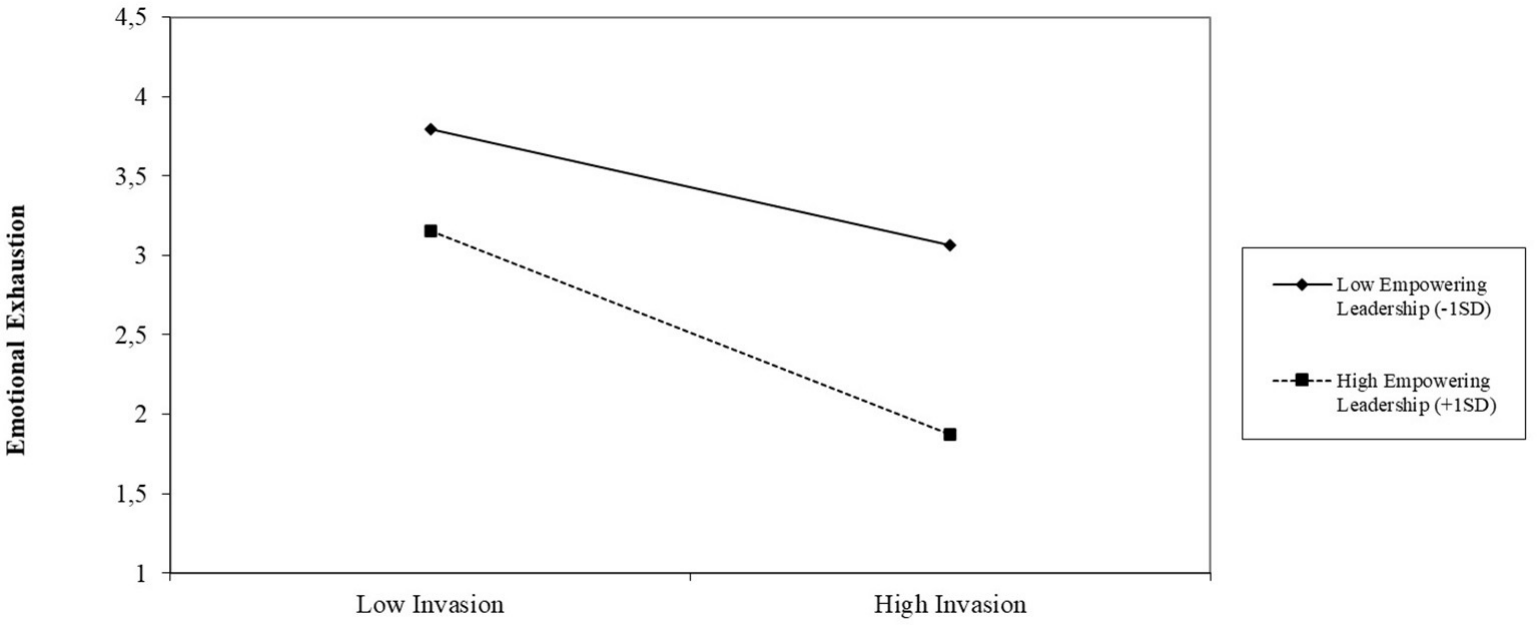
Interaction plot for the relation between techno-complexity and emotional exhaustion under high (+1SD) and low (−1SD) empowering leadership.

Figure 2 displays the interaction plot for the association between techno-invasion and emotional exhaustion under the condition of relatively low (−1SD) and relatively high (+1SD) empowering leadership. The gradient slope for high empowering leadership is −0.345 (*p* < 0.050), which is steeper than the gradient slope for low empowering leadership (gradient slope −0.203, *p* > 0.050). As can be seen from Figure 2, the association between techno-invasion and emotional exhaustion is lower when empowering leadership is relatively high. Figure 3 shows a similar interaction plot for the association between techno-overload, moderated by empowering leadership. The gradient slope for low empowering leadership is 0.324 (*p* < 0.010), which is less steep compared to the gradient slope for high empowering leadership (gradient slope 0.409, *p* < 0.010). Accordingly, Figure 3 demonstrates that the association between techno-overload and emotional exhaustion is stronger when empowering leadership is relatively high. Contrary to H3, the interaction between techno-complexity and empowering leadership was not significant (*B* = 0.062, *p* = 0.051). The bootstrapped results indicated significant conditional indirect effects for techno-overload (−0.052 with 95% CI[−0.086, −0.019], *p* < 0.010) and techno-invasion (−0.072 with 95% CI[−0.118, −0.032], *p* < 0.001) on quality of care delivered, mediated by emotional exhaustion and moderated by empowering leadership. In line with the above results, a significant conditional indirect effect was not observed for techno-complexity (−0.029 with 95% CI[−0.061, 0.002], *p* > 0.050). Therefore, the final hypothesis (H3) was only partially confirmed.

## Discussion

This study examined how technostress (i.e., techno-overload, techno-invasion, techno-complexity) affected the quality of care delivered among childcare workers in the Netherlands, by focusing on the mediating role of emotional exhaustion. In addition, we investigate how these relations were affected by empowering leadership. In line with our hypotheses, our results revealed that two techno-stressors, techno-overload and techno-invasion, stimulate emotional exhaustion among child workers, ultimately reducing the quality of care delivered. However, for techno-complexity, such a relation could not be established. In addition, empowering leadership mitigated the relation between techno-invasion and emotional exhaustion, but modestly strengthened the relation between techno-complexity and emotional exhaustion.

### Theoretical Implications

We make three theoretical contributions. First, this study is an answer to the call of Tarafdar et al. (2015) for more contextual technostress research. By showing that childcare workers are affected by technostress due to the emergent digital transformation of childcare (Yost and Fan, 2014), our study demonstrated that technostress also impacts individual job outcomes beyond the “usual suspects” in high-tech and/or business environments. In care professions, like childcare, ICT has been introduced more sudden compared to, for example, office jobs and blue-collar jobs, where such transformations have occurred more gradually (Califf et al., 2015; Fagerström et al., 2017). Another contribution to the contextualization of technostress is the empirical link with quality of care delivered. While indirect, this link highlights that technostress not only has an impact on general work attitudes, but also affects more complex and context-specific outcomes, like quality of care delivered (Humphries et al., 2014; Alves and Guirardello, 2016). Following Califf and Brooks (2020), context can also help to explain why we found no significant relationship for techno-complexity. In a childcare context, the extent to which childcare workers deal with complex ICT might be more limited as compared to other sectors. The lower mean for techno-complexity compared to the other two stressors seems to confirm that image.

A second contribution of this study is to COR theory by furthering and extending past efforts to integrate this theory, with its origins in the psychological stress literature, with technostress literature (Harris et al., 2015; Goetz and Boehm, 2020). In line with COR theory's idea that technostress imposes a treat to employees' resources at work, we observed that specific techno-stressors depleted childcare workers' job resources, resulting in emotional exhaustion and limiting the efforts childcare workers are able to invest in delivering high-quality care. Furthermore, the discovery of a full mediation also strengthens the idea of a “loss cycle,” central to COR theory. In other words, childcare workers, as committed professionals, do not immediately stop investing in quality of care delivered, but only do so when experiencing emotional exhaustion further depletes their resources, prompting them to save and prioritize their remaining resources. This interpretation is consistent with and extends research demonstrating a mediation of emotional exhaustion between stress and job performance (e.g., Karatepe and Uludag, 2008). It is also in line with empirical studies linking techno-stressors to emotional exhaustion (Brown et al., 2014; Srivastava et al., 2015; Gaudioso et al., 2017) and emotional exhaustion to job performance (Wright and Cropanzano, 1998).

Finally, by examining the role of empowering leadership, this study contributes to the emergent line of research on leadership in the contemporary ICT-infused workplace (Cortellazzo et al., 2019; Bartsch et al., 2020; Iannotta et al., 2020). Indeed, our analyses demonstrate that an empowering leadership style, which encourages autonomy and self-management, can stimulate employees to reduce technostress by engaging in problem solving, making shared decisions and setting boundaries for themselves, hereby replenishing their resource base consistent with the logic of COR theory. In this sense our empirical observations fit calls to integrate COR theory with leadership (Hobfoll et al., 2018; Kim and Beehr, 2021) and resonates with prior empirical studies on technostress(ors) and leadership (e.g., Harris et al., 2015; Windeler et al., 2017; Turel and Gaudioso, 2018). On a more critical note, empowering leadership reduced but did not reverse the effect of techno-invasion on quality of care delivered, mediated by emotional exhaustion. This is in line with the so-called “primacy of loss-hypothesis,” suggesting that employees are more susceptible to stressors than to resources (Hobfoll, 2001). Also, one unexpected finding in our study was that empowering leadership strengthened, rather than reduced the effect of techno-overload on quality of care delivered, mediated by emotional exhaustion. This is in line with Kim et al. (2018), who argued that overwhelmed employees may prefer fewer engaging behaviors by their supervisors. In addition, empowering leaders' emphasis on employee autonomy and responsibility might also create an additional burden and add to existing stressors. Indeed, Sharma and Kirkman (2015) have posited that the combination of workplace stressors and an empowering leadership style might overwhelm employees, thereby weakening the typically positive effects of empowering leadership on employee outcomes. Moreover, higher levels of stress or pressure related to such additional responsibilities might also reduce the empowering initiatives of the leader. Since only a few studies have examined the potential negative effects of empowering leadership (Cheong et al., 2016), future research could further delve into the potential drawbacks such leaders have for technology-related attitudes and behaviors.

### Limitations

Despite the study's strengths, we also wish to point a couple of limitations. First, the cross-sectional nature of the study implies that causal relationships between the variables could not be demonstrated. More importantly, technostress and its coping by employees are suggested to be more dynamic and temporal in nature than reflected in cross-sectional studies (Nimrod, 2018). To better understand fluctuations in technostress and coping among employees, future research could draw on techniques like the experience sampling method (ESM) in which participants react to repeated assessments at different points in time. A recent example is Benlian (2020), but aside from this study, it seems there are limited ESM approaches to technostress. A second limitation is that this that we employed perceptual data from a single source, which might be prone to CMB (George and Pandey, 2017). Nevertheless, the use of single-source perceptual data is warranted when assessing employees' feelings and attitudes, which strongly rely on people's perceptions. Furthermore, the presence of an interaction effect significantly the probability of CMB (Siemsen et al., 2010) and a single factor test showed that CMB presented no major concern to the data and model (Kline, 2011). Third, in this research the focus was on linear relationships. However, recent research also hints at the existence of techno “eustress” (Tarafdar et al., 2019) and non-linear relations of technostress (Srivastava et al., 2015). Finally, there might be limits to the generalizations of the results. While our sample resembled the population of Dutch childcare workers in terms of age and gender (FCB, 2018), our sample might still lack representation on other, non-measured criteria that also affect technostress experiences (e.g., migratory and socio-economic background). Furthermore, our focus on Dutch childcare workers might limit the generalization to countries where the introduction of ICT in childcare and other care professions has been more modest.

### Future Research

Next to addressing the above limitations and continuing the contextualization of technostress within care professions (Califf et al., 2015; Fagerström et al., 2017), future research could address a couple of additional issues. First, scholars suggest that apart from empowering and participative leadership approaches also networked, open and agile leadership present promising leadership approaches through which leaders could replenish ICT-relevant employee resources (Petry, 2018). Second, future research endeavors could investigate which individual and contextual factors foster and/or constrain leaders as technostress buffers. On the one hand, past research that there are significant individual discrepancies in (techno)stress perceptions, associated with differences in age (Nimrod, 2018; Estrada-Muñoz et al., 2020), psychological and personality traits (Lee et al., 2014; Srivastava et al., 2015), as well as behavioral archetypes (González-López et al., 2021) and boundary preferences (Gadeyne et al., 2018; Bauwens et al., 2020), which could determine the effectiveness of leaders as resource replenishers for technostress. Therefore, we suggest future research to account for these variables in their design. On the other hand, leaders' supporting role could also be hindered or fast-tracked by organizational climates (Turel and Gaudioso, 2018) or the presence of other leaders within the organizational hierarchy (Batistič et al., 2017). For example, in larger organizations, decisions concerning technology are often taken not by the direct supervisor, but at higher leadership levels. Consequentially, the leader behaviors supervisors undertake to address technological challenges might also be contingent or shaped by the leader behaviors of those in the higher echelons of the organization. Beyond the scope of leaders, studies could also adopt a different range of techno-stressors. For example, Fischer et al. (2021) very recently developed a scale which distinguishes between 15 ICT-related stressors. Conversely, Hu et al. (2021) a little while ago challenged our thinking about techno-stressors altogether by suggesting to “integrate the research on ICT and employee health and well-being to ‘clean up' ICT terminologies and measures” (Hu et al., 2021, p. 22). This suggests that conceptual development in technostress literature is still ongoing and that as this literature evolves, so will our understanding of technostress and its link with leaders and employee outcomes. In other words: brace yourself for exciting times ahead!

### Practical Implications

This study has a number of implications, in particular for childcare and other care organizations. Since childcare workers constitute an employee group whose work-related health is continuously at risk (Decker et al., 2002; Løvgren, 2016; Koch et al., 2017) and the use of ICT in the sector is likely to continue its accelerated pace over the next few years, it is important that childcare organizations are aware of this risk and acknowledge the potential implications of ICT. To mitigate technostress, COR theory presents a useful tool to organizations, arguing that interventions should strengthen employee's resource base (Hobfoll and Freedy, 2017). In this regard, our findings suggest such interventions could take the form of leadership interventions, geared at non-directive leader behaviors that strive to foster employees' autonomy, participative decision making, problem-solving behavior (Arnold et al., 2000; Ahearne et al., 2005; Audenaert and Decramer, 2016). Such leader behaviors could strengthen employees' resource base, stimulating employees to broaden their scope of potential solutions for given problems by exploring opportunities and alternatives, ultimately making them more resilient to technostress and preserving their well-being and care quality. However, leaders and organizations also have to remain vigilant, as in certain situations such leader behaviors could also overburden employees.

## Data Availability Statement

The raw data supporting the conclusions of this article will be made available by the authors, without undue reservation.

## Ethics Statement

The studies involving human participants were reviewed and approved by Ethics Review Board (ERB), School of Social and Behavioral Sciences, Tilburg University (no. EC-2019.76). The patients/participants provided their written informed consent to participate in this study.

## Author Contributions

RB: conceptualization, data collection, formal analysis, writing—original draft, and project administration. MD: conceptualization, data collection, formal analysis, and writing—original draft. JV and MC: writing—original draft, review, and editing. All authors contributed to the article and approved the submitted version.

## Conflict of Interest

The authors declare that the research was conducted in the absence of any commercial or financial relationships that could be construed as a potential conflict of interest.

## Footnotes

^1^https://www.rijksoverheid.nl/onderwerpen/kwaliteit-van-de-zorg/kwaliteitseisen-zorginstellingen

^2^https://www.landelijkregisterkinderopvang.nl/

^3^https://www.rijksoverheid.nl/onderwerpen/coronavirus-covid-19/onderwijs-en-kinderopvang/ouders

## References

[B1] Ahearne M. Mathieu J. Rapp A. (2005). To empower or not to empower your sales force? An empirical examination of the influence of leadership empowerment behavior on customer satisfaction and performance. J. Appl. Psychol. 90, 945–955. 10.1037/0021-9010.90.5.945 16162066

[B2] Aiken L. H. Clarke S. P. Sloane D. M. (2002), Hospital staffing, organization, quality of care: cross-national findings. Int. J. Qual. Health Care 14, 5–14. 10.1093/intqhc/14.1.5 11871630

[B3] Alves D. F. S. Guirardello E. B. (2016). Safety climate, emotional exhaustion and job satisfaction among Brazilian paediatric professional nurses. Int. Nurs. Rev. 63, 328–335. 10.1111/inr.12276 27265871

[B4] Arnold J. A. Arad S. Rhoades J. A. Drasgow F. (2000). The empowering leadership questionnaire: the construction and validation of a new scale for measuring leader behaviors. J. Organ. Behav. 21, 249–269. 10.1002/(SICI)1099-1379(200005)21:3<249::AID-JOB10>3.0.CO;2-#

[B5] Audenaert M. Decramer A. (2016). When empowering leadership fosters creative performance: the role of problem-solving demands and creative personality. J. Manage. Organ. 24, 4–18. 10.1017/jmo.2016.20

[B6] Audenaert M. George B. Bauwens R. Decuypere A. Descamps A.-M. Muylaert J.. (2020). Empowering leadership, social support, and job crafting in public organizations: a multilevel study. Public Pers. Manage. 49, 367–392. 10.1177/0091026019873681

[B7] Ayyagari R. Grover V. Purvis R. (2011). Technostress: Technological Antecedents and Implications. MIS Q. 35, 831–858. 10.2307/41409963

[B8] Bartsch S. Weber E. Büttgen M. Huber A. (2020). Leadership matters in crisis-induced digital transformation: how to lead service employees effectively during the COVID-19 pandemic. J. Serv. Manage. 32, 71–85. 10.1108/JOSM-05-2020-0160

[B9] Batistič S. Cerne M. Vogel B. (2017). Just how multi-level is leadership research? A document co-citation analysis 1980-2013 on leadership constructs and outcomes. Leadership Q. 28, 86–103. 10.1016/j.leaqua.2016.10.007

[B10] Bauwens R. Batistič S. Kilroy S. Nijs S. (2021). New kids on the block? A bibliometric analysis of emerging COVID-19-trends in leadership research. J. Leadership Organ. Stud. 10.1177/1548051821997406. [Epub ahead of print] PMC8990571 35516092

[B11] Bauwens R. Meyfroodt K. (2021). Debate: Towards a more comprehensive understanding of ritualized bureaucracy in digitalized public organizations. Public Money Manage. 41, 281–282. 10.1080/09540962.2021.1884349

[B12] Bauwens R. Muylaert J. Clarysse E. Audenaert M. Decramer A. (2020). Teachers' acceptance and use of digital learning environments after hours: implications for work-life balance and the role of integration preference. Comput. Hum. Behav. 112:106479. 10.1016/j.chb.2020.106479

[B13] Benlian A. (2020). A daily field investigation of technology-driven spillovers from work to home. MIS Q. 44, 1259–1300. 10.25300/MISQ/2020/14911/

[B14] Bradley M. Chahar P. (2020). Burnout of healthcare providers during COVID-19. Cleveland Clin. J. Med. 10.3949/ccjm.87a.ccc051 32606049

[B15] Brod C. (1982). Managing technostress: optimizing the use of computer technology. Pers. J. 61, 753–757. 10258012

[B16] Brooks S. Califf C. (2017). Social media-induced technostress: its impact on the job performance of it professionals and the moderating role of job characteristics. Comput. Netw. 114, 143–153. 10.1016/j.comnet.2016.08.020

[B17] Brown R. Duck J. Jimmieson N. (2014). E-mail in the workplace: the role of stress appraisals and normative response pressure in the relationship between e-mail stressors and employee strain. Int. J. Stress Manage. 21, 325–347. 10.1037/a0037464

[B18] Califf C. Sarker S. Sarker S. Fitzgerald C. (2015). The bright and dark sides of technostress: an empirical study of healthcare workers in Proceedings of ICIS 2015 AIS (Chair) (Fort Worth, TX).

[B19] Califf C. B. Brooks S. (2020). An empirical study of techno-stressors, literacy facilitation, burnout, and turnover intention as experienced by K-12 teachers. Comput. Educ. 157, 103971. 10.1016/j.compedu.2020.103971

[B20] CBS (2020). Monitor Arbeid, Zorg en Kinderopvang 2019 [Monitor Labor, Care and Childcare 2019]. Retrieved from: https://www.cbs.nl/-/media/_excel/2020/18/monitor-arbeid-zorg-kinderopvang-2019.xlsx

[B21] Cheong M. Spain S. M. Yammarino F. J. Yun S. (2016). Two faces of empowering leadership: enabling and burdening. Leadership Q. 27, 602–616. 10.1016/j.leaqua.2016.01.006

[B22] Cheong M. Yammarino F. J. Dionne S. D. Spain S. M. Tsai C.-Y. (2019). A review of the effectiveness of empowering leadership. Leadership Q. 30, 34–58. 10.1016/j.leaqua.2018.08.005

[B23] Cortellazzo L. Bruni E. Zampieri R. (2019). The role of leadership in a digitalized world: a review. Front. Psychol. 10:1938. 10.3389/fpsyg.2019.01938 31507494 PMC6718697

[B24] Coun M. Peters P. Blomme R. J. Schaveling J. (2021). To empower or not to empower, that's the question. Using an empowerment process approach to explain employees' workplace proactivity. Int. J. Hum. Resour. Manage. 10.1080/09585192.2021.1879204. [Epub ahead of print]

[B25] Decker J. T. Bailey T. L. Westergaard N. (2002). Burnout among childcare workers. Resident. Treat. Child. Youth 19, 61–77. 10.1300/J007v19n04_04

[B26] Denissen M. (2020). Technostress, emotional exhaustion and quality of care in childcare organizations (Master's thesis). Tilburg University, Tilburg.

[B27] Dinh J. E. Lord R. G. Gardner W. L. Meuser J. D. Liden R. C. Hu J. (2014). Leadership theory and research in the new millennium: current theoretical trends and changing perspectives. Leadership Q. 25, 36–62. 10.1016/j.leaqua.2013.11.005

[B28] Estrada-Muñoz C. Castillo D. Vega-Muñoz A. Boada-Grau J. (2020). Teacher technostress in the Chilean School system. Int. J. Environ. Res. Public Health 17:5280. 10.3390/ijerph17155280 32707973 PMC7432078

[B29] Fagerström C. Tuvesson H. Axelsson L. Nilsson L. (2017). The role of ICT in nursing practice: an integrative literature review of the Swedish context. Scand. J. Caring Sci. 31, 434–448. 10.1111/scs.12370 27507258

[B30] FCB (2018). Factsheet Technologische Ontwikkelingen [Factsheet Technological Developments]. Retrieved from: https://www.kinderopvang-werkt.nl/sites/fcb_kinderopvang/files/2020-11/factsheet_technologie_wge_2018_ko.pdf

[B31] Feng Q. Song Q. Zhang L. Zheng S. Pan J. (2020). Integration of moderation and mediation in a latent variable framework: a comparison of estimation approaches for the second-stage moderated mediation model. Front. Psychol. 11:2167. 10.3389/fpsyg.2020.02167 33013556 PMC7511593

[B32] Fischer T. Reuter M. Riedl R. (2021). The digital stressors scale: development and validation of a new survey instrument to measure digital stress perceptions in the workplace context. Front. Psychol. 12:646. 10.3389/fpsyg.2021.607598 33776836 PMC7994533

[B33] Gadeyne N. Verbruggen M. Delanoeije J. De Cooman R. (2018). All wired, all tired? Work-related ICT-use outside work hours and work-to-home conflict: the role of integration preference, integration norms and work demands. J. Voc. Behav. 107, 86–99. 10.1016/j.jvb.2018.03.008

[B34] Gardner W. L. Lowe K. B. Meuser J. D. Noghani F. Gullifor D. P. Cogliser C. C. (2020). The leadership trilogy: a review of the third decade of the leadership quarterly. Leadership Q. 31:101379. 10.1016/j.leaqua.2019.101379

[B35] Gaudioso F. Turel O. Galimberti C. (2017). The mediating roles of strain facets and coping strategies in translating techno-stressors into adverse job outcomes. Comput. Hum. Behav. 69, 189–196. 10.1016/j.chb.2016.12.041

[B36] George B. Pandey S. K. (2017). We know the Yin-but where is the Yang? Toward a balanced approach on common source bias in public administration scholarship. Rev. Public Pers. Admin. 37, 245–270. 10.1177/0734371X17698189 29046599 PMC5633037

[B37] Ghislieri C. Emanuel F. Molino M. Cortese C. G. Colombo L. (2017). New technologies smart, or harm work-family boundaries management? Gender differences in conflict and enrichment using the JD-R theory. Front. Psychol. 8:1070. 10.3389/fpsyg.2017.01070 28713300 PMC5492914

[B38] Goetz T. M. Boehm S. A. (2020). Am I outdated? The role of strengths use support and friendship opportunities for coping with technological insecurity. Comput. Hum. Behav. 107:106265. 10.1016/j.chb.2020.106265

[B39] González-López Ó. R. Buenadicha-Mateos M. Sánchez-Hernández M. I. (2021). Overwhelmed by technostress? Sensitive archetypes and effects in times of forced digitalization. Int. J. Environ. Res. Public Health 18:4216. 10.3390/ijerph18084216 33923407 PMC8074205

[B40] Harris K. J. Harris R. B. Carlson J. R. Carlson D. S. (2015). Resource loss from technology overload and its impact on work-family conflict: can leaders help? Comput. Hum. Behav. 50, 411–417. 10.1016/j.chb.2015.04.023

[B41] Hill N. S. Bartol K. M. (2016). Empowering leadership and effective collaboration in geographically dispersed teams. Pers. Psychol. 69, 159–198. 10.1111/peps.12108

[B42] Hobfoll S. E. (2001). The influence of culture, community, and the nested-self in the stress process: advancing conservation of resources theory. Appl. Psychol. 50, 337–421. 10.1111/1464-0597.00062

[B43] Hobfoll S. E. Freedy J. (2017). Conservation of resources: a general stress theory applied to burnout, in Professional Burnout, eds Schaufeli W. Maslach C. Marek T. (London: Routledge), 115–129. 10.4324/9781315227979-9

[B44] Hobfoll S. E. Halbesleben J. Neveu J. P. Westman M. (2018). Conservation of resources in the organizational context: the reality of resources and their consequences. Annu. Rev. Organ. Psychol. Organ. Behav. 5, 103–128. 10.1146/annurev-orgpsych-032117-104640

[B45] Hu X. Park Y. A. Day A. Barber L. K. (2021). Time to disentangle the information and communication technology (ICT) constructs: developing a taxonomy around ICT use for occupational health research. Occupat. Health Sci. 10.1007/s41542-021-00085-6 33748406 PMC7962926

[B46] Humphries N. Morgan K. Catherine Conry M. McGowan Y. Montgomery A. McGee H. (2014). Quality of care and health professional burnout: Narrative literature review. Int. J. Health Care Qual. Assur. 27, 293–307. 10.1108/IJHCQA-08-2012-0087 25076604

[B47] Iannotta M. Meret C. Marchetti G. (2020). Defining leadership in smart working contexts: a concept synthesis. Front. Psychol. 11:2448. 10.3389/fpsyg.2020.556933 33041921 PMC7525207

[B48] Jorgensen T. D. Pornprasertmanit S. Schoemann A. Rosseel Y. Miller P. Quick C.. (2018). Package 'semTools'. Available online at: https://cran.r-project.org/web/packages/semTools/semTools.pdf

[B49] Karatepe O. M. Uludag O. (2008). Role stress, burnout and their effects on frontline hotel employees' job performance: evidence from Northern Cyprus. Int. J. Tourism Res. 10, 111–126. 10.1002/jtr.645

[B50] Kilroy S. Bosak J. Flood P. C. Peccei R. (2020). Time to recover: the moderating role of psychological detachment in the link between perceptions of high-involvement work practices and burnout. J. Bus. Res. 108, 52–61. 10.1016/j.jbusres.2019.10.012

[B51] Kim M. Beehr T. A. (2020). The long reach of the leader: can empowering leadership at work result in enriched home lives? J. Occupat. Health Psychol. 25, 203–213. 10.1037/ocp0000177 31999139

[B52] Kim M. Beehr T. A. (2021). The power of empowering leadership: Allowing and encouraging followers to take charge of their own jobs. The International Journal of Human Resource Management. 39(9), 1865–1898. 10.1080/09585192.2019.1657166

[B53] Kim M. Beehr T. A. Prewett M. S. (2018). Employee responses to empowering leadership: a meta-analysis. J. Leadership Organ. Stud. 25, 257–276. 10.1177/1548051817750538

[B54] Kline R. B. (2011). Principles and Practice of Structural Equation Modeling, 3rd Edn. New York, NY: Guilford Press.

[B55] Koch P. Kersten J. F. Stranzinger J. Nienhaus A. (2017). The effect of effort-reward imbalance on the health of childcare workers in Hamburg: a longitudinal study. J. Occupat. Med. Toxicol. 12, 1–9. 10.1186/s12995-017-0163-8 28670329 PMC5485678

[B56] Kroon B. van de Voorde K. van Veldhoven M. (2009). Cross-level effects of high-performance work practices on burnout. Pers. Rev. 38, 509–525. 10.1108/00483480910978027

[B57] La Torre G. Esposito A. Sciarra I. Chiappetta M. (2019). Definition, symptoms and risk of techno-stress: a systematic review. Int. Arch. Occupat. Environ. Health 92, 13–35. 10.1007/s00420-018-1352-1 30196317

[B58] Lee Y.-K. Chang C.-T. Lin Y. Cheng Z.-H. (2014). The dark side of smartphone usage: psychological traits, compulsive behavior and technostress. Comput. Hum. Behav. 31, 373–383. 10.1016/j.chb.2013.10.047

[B59] Liu L. Yang C. Huang D. (2021). How do empowered leaders influence the job satisfaction of kindergarten teachers in China? Evidence from mediation analysis. Front. Psychol. 11:3694. 10.3389/fpsyg.2020.586943 33584421 PMC7874125

[B60] Løvgren M. (2016). Emotional exhaustion in day-care workers. Eur. Early Childhood Educ. Res. J. 24, 157–167. 10.1080/1350293X.2015.1120525

[B61] Lutz S. Schneider F. M. Vorderer P. (2020). On the downside of mobile communication: an experimental study about the influence of setting-inconsistent pressure on employees' emotional well-being. Comput. Hum. Behav. 105:106216. 10.1016/j.chb.2019.106216

[B62] Maslach C. Jackson S. E. Leiter M. P. Schaufeli W. B. Schwab R. L. (1986), Maslach Burnout Inventory. Palo Alto, CA: Consulting Psychologists Press.

[B63] Maslach C. Schaufeli W. B. Leiter M. P. (2001). Job burnout. Annu. Rev. Psychol. 52, 397–422. 10.1146/annurev.psych.52.1.397 11148311

[B64] Molino M. Ingusci E. Signore F. Manuti A. Giancaspro M. L. Russo V.. (2020). Wellbeing costs of technology use during covid-19 remote working: an investigation using the Italian translation of the technostress creators scale. Sustainability 12:5911. 10.3390/su12155911

[B65] Nimrod G. (2018). Technostress: measuring a new threat to well-being in later life. Aging Mental Health 22, 1086–1093. 10.1080/13607863.2017.1334037 28562064

[B66] Parker S. K. Grote G. (2020). Automation, algorithms, and beyond: why work design matters more than ever in a digital world. Appl. Psychol. 10.1111/apps.12241

[B67] Pearce C. L. Sims H. P. (2002). Vertical versus shared leadership as predictors of the effectiveness of change management teams: an examination of aversive, directive, transactional, transformational, and empowering leader behaviors. Group Dyn. Theory Res. Pract. 6, 172–197. 10.1037/1089-2699.6.2.172

[B68] Penado Abilleira M. Rodicio-García M.-L. Ríos-de Deus M. P. Mosquera-González M. J. (2021). Technostress in Spanish University teachers during the COVID-19 pandemic. Front. Psychol. 12:617650. 10.3389/fpsyg.2021.617650 33732187 PMC7959820

[B69] Petry T. (2018), Digital leadership, in Knowledge Management in Digital Change, eds North K. Maier R Haas O. (Cham: Springer), 209–218. 10.1007/978-3-319-73546-7_12.

[B70] Ragu-Nathan T. S. Tarafdar M. Ragu-Nathan B. S. Tu Q. (2008). The consequences of technostress for end users in organizations: conceptual development and empirical validation. Inform. Syst. Res. 19, 417–433. 10.1287/isre.1070.0165

[B71] Rosseel Y. (2012). lavaan: an R package for structural equation modeling. J. Stat. Softw. 48, 1–36. 10.18637/jss.v048.i0225601849

[B72] Salanova M. Llorens S. Cifre E. (2013). The dark side of technologies: technostress among users of information and communication technologies. Int. J. Psychol. 48, 422–436. 10.1080/00207594.2012.680460 22731610

[B73] Schaufeli W. B. (2015). Engaging leadership in the job demands-resources model. Career Dev. Int. 20, 446–463. 10.1108/CDI-02-2015-0025

[B74] Schaufeli W. B. Leiter M. P. Maslach C. Jackson S. E. (1996). Maslach Burnout Inventory - General Survey (MBI-GS). Palo Alto, CA: Mind Garden.

[B75] Schlachter S. McDowall A. Cropley M. Inceoglu I. (2018). Voluntary work-related technology use during non-work time: a narrative synthesis of empirical research and research agenda. Int. J. Manage. Rev. 20, 825–846. 10.1111/ijmr.12165

[B76] Sharma P. N. Kirkman B. L. (2015). Leveraging leaders. Group Organ. Manage. 40, 193–237. 10.1177/1059601115574906

[B77] Shu Q. Tu Q. Wang K. (2011). The impact of computer self-efficacy and technology dependence on computer-related technostress: a social cognitive theory perspective. Int. J. Hum. Comput. Interact. 27, 923–939. 10.1080/10447318.2011.555313

[B78] Siemsen E. Roth A. Oliveira P. (2010). Common method bias in regression models with linear, quadratic, and interaction effects. Organ. Res. Methods 13, 456–476. 10.1177/1094428109351241

[B79] Spagnoli P. Molino M. Molinaro D. Giancaspro M. L. Manuti A. Ghislieri C. (2020). Workaholism and technostress during the COVID-19 emergency: the crucial role of the leaders on remote working. Front. Psychol. 11:3714. 10.3389/fpsyg.2020.620310 33424730 PMC7786603

[B80] Srivastava S. C. Chandra S. Shirish A. (2015). Technostress creators and job outcomes: theorising the moderating influence of personality traits. Inform. Syst. J. 25, 355–401. 10.1111/isj.12067

[B81] Tarafdar M. Cooper C. L. Stich J. F. (2019). The technostress trifecta - techno eustress, techno distress and design: theoretical directions and an agenda for research. Inform. Syst. J. 29, 6–42. 10.1111/isj.12169

[B82] Tarafdar M. Pullins E. B. Ragu-Nathan T. S. (2015). Technostress: negative effect on performance and possible mitigations. Inform. Syst. J. 25, 103–132. 10.1111/isj.12042

[B83] Tarafdar M. Tu Q. Ragu-Nathan T. S. (2010). Impact of technostress on end-user satisfaction and performance. J. Manage. Inform. Syst. 27, 303–334. 10.2753/MIS0742-1222270311

[B84] Turel O. Gaudioso F. (2018). Technostressors, distress and strain: the roles of leadership and competitive climates. Cogn. Technol. Work 20, 309–324. 10.1007/s10111-018-0461-7

[B85] Wang W. Sakata K. Komiya A. Li Y. (2020). What makes employees' work so stressful? Effects of vertical leadership and horizontal management on employees' stress. Front. Psychol. 11:340. 10.3389/fpsyg.2020.00340 32265768 PMC7096576

[B86] Wang X. Tan S. C. Li L. (2020). Technostress in University students' technology-enhanced learning: An investigation from multidimensional person-environment misfit. Comput. Hum. Behav. 105:106208. 10.1016/j.chb.2019.106208

[B87] Weil M. M. Rosen L. D. (1997). Technostress: Coping with Technology@ Work@ Home@ Play. New York, NY: Wiley.

[B88] Windeler J. B. Maruping L. Venkatesh V. (2017). Technical systems development risk factors: the role of empowering leadership in lowering developers' stress. Inform. Syst. Res. 28, 775–796. 10.1287/isre.2017.0716

[B89] Wright T. A. Cropanzano R. (1998). Emotional exhaustion as a predictor of job performance and voluntary turnover. J. Appl. Psychol. 83, 486–493. 10.1037/0021-9010.83.3.486 9648526

[B90] Yost H. Fan S. (2014). Social media technologies for collaboration and communication: perceptions of childcare professionals and families. Austral. J. Early Childhood 39, 36–41. 10.1177/183693911403900206

[B91] Zhang Y. LePine J. A. Buckman B. R. Wei F. (2014). It's not fair … or is it? The role of justice and leadership in explaining work stressor-job performance relationships. Acad. Manage. J. 57, 675–697. 10.5465/amj.2011.1110

